# Supplementary material for: Epidemiological Trends of Coronavirus Disease 2019 in China
Source: Front Med (Lausanne). 2020 May 29;7:259. doi: 10.3389/fmed.2020.00259 (PMC7273499; doi:10.3389/fmed.2020.00259)
Supplement: Supplementary file 1 [file Data_Sheet_1.DOCX]

# *Supplementary Material*

Data of the Hubei provinces in China are based on daily briefings from Health Commission of Hubei Province.(1) Data of the Guangdong province in China are based on daily briefings from Health Commission of Guangdong Province.(2) Data of the Henan province in China are based on daily briefings from Health Commission of Henan Province.(3) Data of the Zhejiang province in China are based on daily briefings from Health Commission of Zhejiang Province.(4) Data of the Hunan province in China are based on daily briefings from Health Commission of Hunan Province.(5) Data of the Anhui province in China are based on daily briefings from Health Commission of Anhui Province.(6) Data of the Jiangxi province in China are based on daily briefings from Health Commission of Jiangxi Province.(7) Data of the Shandong province in China are based on daily briefings from Health Commission of Shandong Province.(8) Data of the Jiangsu province in China are based on daily briefings from Jiangsu Commission of Health.(9) Data of the Chongqing Municipal in China are based on daily briefings from Chongqing Municipal Health Commission.(10) Data of the Sichuan province in China are based on daily briefings from Health Commission of Sichuan Province.(11) Data of the Heilongjiang province in China are based on daily briefings from Health Commission of Heilongjiang Province.(12) Data of the Beijing Municipal in China are based on daily briefings from Beijing Municipal Health Commission.(13) Data of the Shanghai Municipal in China are based on daily briefings from Beijing Municipal Health Commission.(14) Data of the Hebei province in China are based on daily briefings from Health Commission of Hebei Province.(15) Data of the Fujian province in China are based on daily briefings from Fujian Provincial Health Commission.(16) Data of the Guangxi province in China are based on daily briefings from Health Commission of Guangxi Province.(17) Data of the Shaanxi province in China are based on daily briefings from Health Commission of Shaanxi Province.(18) Data of the Yunnan province in China are based on daily briefings from Health Commission of Yunnan Province.(19) Data of the Hainan province in China are based on daily briefings from Health Commission of Hainan Province.(20) Data of the Guizhou province in China are based on daily briefings from Health Commission of Guizhou Province.(21) Data of the Shanxi province in China are based on daily briefings from Health Commission of Shanxi Province.(22) Data of the Tianjin Municipal in China are based on daily briefings from Tianjin Municipal Health Commission.(23) Data of the Liaoning province in China are based on daily briefings from Health Commission of Liaoning Province.(24) Data of the Gansu province in China are based on daily briefings from Health Commission of Gansu Province.(25) Data of the Jilin province in China are based on daily briefings from Health Commission of JilinProvince.(26) Data of the Xinjiang province in China are based on daily briefings from Health Commission of Xinjiang Province.(27) Data of the Inner Mongolia province in China are based on daily briefings from Health Commission of Inner Mongolia Province.(28) Data of the Ningxia province in China are based on daily briefings from Health Commission of Ningxia Province.(29) Data of the Qinghai province in China are based on daily briefings from Health Commission of Qinghai Province.(30) Data of the Tibet province in China are based on daily briefings from Health Commission of Tibet Province.(31)

# Supplementary Reference

1. Health Commission of Hubei Province's briefing on the pneumonia epidemic situation. [cited 2020 February 24]. Available from: <http://wjw.hubei.gov.cn/bmdt/ztzl/fkxxgzbdgrfyyq/xxfb/>.

2. Health Commission of Guangdong Province's briefing on the pneumonia epidemic situation. [cited 2020 February 20]. Available from: <http://wsjkw.gd.gov.cn/zwyw_yqxx/index.html>.

3. Health Commission of Henan Province's briefing on the pneumonia epidemic situation. [cited 2020 February 24]. Available from: <http://www.hnwsjsw.gov.cn/channels/858.shtml>.

4. Health Commission of Zhejiang Province's briefing on the pneumonia epidemic situation. [cited 2020 February 24]. Available from: <http://www.zjwjw.gov.cn/col/col1202101/index.html?uid=4978845&pageNum=2>.

5. Health Commission of Hunan Province's briefing on the pneumonia epidemic situation. [cited 2020 February 24]. Available from: <http://wjw.hunan.gov.cn/wjw/qwfb/yqfkgz_list.html>.

6. Health Commission of Anhui Province's briefing on the pneumonia epidemic situation. [cited 2020 February 24]. Available from: <http://wjw.ah.gov.cn/ztzl/xxgzbdfyyqfk/xxfb/index.html>.

7. Health Commission of Jiangxi Province's briefing on the pneumonia epidemic situation. [cited 2020 February 24]. Available from: <http://hc.jiangxi.gov.cn/ztxx/xxgzbdgrdfyyqfk/yqtb/index.shtml>.

8. Health Commission of Shandong Province's briefing on the pneumonia epidemic situation. [cited 2020 February 24]. Available from: <http://wsjkw.shandong.gov.cn/ztzl/rdzt/qlzhfkgz/tzgg/>.

9. Jiangsu Commission of Health's briefing on the pneumonia epidemic situation. [cited 2020 February 24]. Available from: <http://wjw.jiangsu.gov.cn/col/col7290/index.html>.

10. Chongqing Municipal Health Commission's briefing on the pneumonia epidemic situation. [cited 2020 February 24]. Available from: <http://wsjkw.cq.gov.cn/ztzl_242/qlzhxxgzbdfyyqfkgz/yqtb/>.

11. Health Commission of Sichuan Province's briefing on the pneumonia epidemic situation. [cited 2020 February 24]. Available from: <http://wsjkw.sc.gov.cn/scwsjkw/gzbd01/ztwzlmgl.shtml>.

12. Health Commission of Heilongjiang Province's briefing on the pneumonia epidemic situation. [cited 2020 February 24]. Available from: <http://wsjkw.hlj.gov.cn/index.php/Home/Zwgk/all/typeid/42>.

13. Beijing Municipal Health Commission's briefing on the pneumonia epidemic situation. [cited 2020 February 24]. Available from: <http://wjw.beijing.gov.cn/wjwh/ztzl/xxgzbd/gzbdyqtb/index.html>.

14. Shanghai Municipal Health Commission's briefing on the pneumonia epidemic situation. [cited 2020 February 24]. Available from: <http://wsjkw.sh.gov.cn/yqtb/index.html>.

15. Health Commission of Hebei Province's briefing on the pneumonia epidemic situation. [cited 2020 February 24]. Available from: <http://wsjkw.hebei.gov.cn/list/zt_gzfy_3714.html>.

16. Fujian Provincial Health Commission's briefing on the pneumonia epidemic situation. [cited 2020 February 24]. Available from: <http://wjw.fujian.gov.cn/ztzl/gzbufk/yqtb/>.

17. Health Commission of Guangxi Province's briefing on the pneumonia epidemic situation. [cited 2020 February 24]. Available from: <http://wsjkw.gxzf.gov.cn/zhuantiqu/ncov/ncovyqtb/>.

18. Health Commission of Shaanxi Province's briefing on the pneumonia epidemic situation. [cited 2020 February 24]. Available from: <http://sxwjw.shaanxi.gov.cn/col/col863/index.html?uid=572&pageNum=2>.

19. Health Commission of Yunnan Province's briefing on the pneumonia epidemic situation. [cited 2020 February 24]. Available from: <http://ynswsjkw.yn.gov.cn/wjwWebsite/web/col?id=UU158123169468495677&cn=yqfb&pcn=ztlm&pid=UU145102906505319731>.

20. Health Commission of Hainan Province's briefing on the pneumonia epidemic situation. [cited 2020 February 24]. Available from: <http://wst.hainan.gov.cn/swjw/rdzt/yqfk/index.html>.

21. Health Commission of Guizhou Province's briefing on the pneumonia epidemic situation [cited 2020 February 24]. Available from: <http://www.gzhfpc.gov.cn/xwzx_500663/yqtb/>.

22. Health Commission of Shanxi Province's briefing on the pneumonia epidemic situation. [cited 2020 February 24]. Available from: <http://wjw.shanxi.gov.cn/xingfew/index.hrh>.

23. Tianjin Municipal Health Commission's briefing on the pneumonia epidemic situation. [cited 2020 February 24]. Available from: <http://wsjk.tj.gov.cn/col/col87/index.html>.

24. Health Commission of Liaoning Province's briefing on the pneumonia epidemic situation. [cited 2020 February 24]. Available from: <http://wsjk.ln.gov.cn/wst_zdzt/xxgzbd/yqtb/>.

25. Health Commission of Gansu Province's briefing on the pneumonia epidemic situation. [cited 2020 February 24]. Available from: <http://wsjk.gansu.gov.cn/channel/11218/index.html>.

26. Health Commission of Jilin Province's briefing on the pneumonia epidemic situation. [cited 2020 February 24]. Available from: <http://www.jl.gov.cn/szfzt/jlzxd/yqtb/>.

27. Health Commission of Xinjiang Province's briefing on the pneumonia epidemic situation. [cited 2020 February 24]. Available from: <http://www.xjhfpc.gov.cn/ztzl/fkxxgzbdfygz/yqtb.htm>.

28. Health Commission of Inner Mongolia Province's briefing on the pneumonia epidemic situation. [cited 2020 February 24]. Available from: <http://wjw.nmg.gov.cn/ztlm/2016n/xxgzbdgrdfyyqfk/yqtb/index.shtml>.

29. Health Commission of Ningxia Hui Autonomous Region's briefing on the pneumonia epidemic situation. [cited 2020 February 24]. Available from: <http://wsjkw.nx.gov.cn/yqfkdt/yqsd1.htm>.

30. Health Commission of Qinghai Province's briefing on the pneumonia epidemic situation. [cited 2020 February 24]. Available from: <https://wsjkw.qinghai.gov.cn/ztbd/yqjk/yqtb/index.html>.

31. Health Commission of Tibet Province's briefing on the pneumonia epidemic situation. [cited 2020 February 24]. Available from: <http://wjw.xizang.gov.cn/xwzx/wsjkdt/>.
